# Drug Brand Response and Its Impact on Compliance and Efficacy in Depression Patients

**DOI:** 10.3389/fphar.2016.00540

**Published:** 2017-01-10

**Authors:** Mingming Li, Jian Cai, Ping Zhang, Chunhua Fei, Feng Xu

**Affiliations:** ^1^Department of Clinical Pharmacy, Fengxian Hospital, Southern Medical UniversityShanghai, China; ^2^Department of Mood Disorder, Fengxian Mental Health CenterShanghai, China

**Keywords:** depression patient, brand response, drug switch, medication compliance, efficacy, side effect

## Abstract

**Introduction:** Patient's response to drug brand is a comprehensive physiological and psychological effect which might impact the compliance and efficacy of drugs. Whether the therapeutic outcome altered on patients with brand response after they experience drug switch is not clear.

**Methods:** 459 outpatients with mild-to-moderate depression were divided into the imported (joint venture) drug group and the domestic drug group according to their current drug application. Two groups of patients were assessed by drug brand preference questionnaire and medication compliance questionnaire. Patients with brand preference in imported (joint venture) drugs group received rational use of limited medical resource and pharmacoeconomics education, and then switched with domestic drug for 8 weeks. Safety and efficacy were evaluated both before and after the drug switch.

**Results:** Overall, there were 27% of patients in imported drug group and 35% of patients in domestic drug group have brand response, respectively. About 2/3 patients in both groups showed low or no brand response. The compliance was similar in both groups with no significant difference (6.04 ± 2.08 vs. 4.74 ± 2.13, respectively, *P* > 0.05). The efficacy of imported drug group was significantly better than of the domestic drug group. Correlation analysis showed that in imported (joint venture) drugs group, medication compliance was closely related with brand response, but negatively correlated with age and duration. In domestic drugs group, medication compliance was independent of brand response, but closely related with education, age, and duration. After drug switch with domestic drug on patients with brand response, patients continued to maintain good antidepressant effect, and no severe adverse reaction occurred.

**Conclusion:** The results suggested that domestic drugs switch might be feasible for patients using imported drugs with brand response, while providing patients with rational use of drug education and psychological support. The medical staff should focus on medication education to help patients make better use of limited medical resources.

## Introduction

Patient's response to treatment is a comprehensive physiological and psychological effect (Moerman, 2002, 2006; Moerman and Jonas, 2002). The treatment covers very wide, from drug brand, dosage forms, preparation color, the amount of oral tablet or capsule, injection volume, to health professionals' appearance, manner, behavior, facial expression and tone of voice. In China, since we have a large number of drug manufactures, there are thousands of drugs available covering domestic drug, imported (joint venture) dugs, as well as originators. Usually we refer to imported (joint venture) dugs and originators as brand drugs, while domestic drugs are generics (Huttin, 1994; Sun et al., 2008). Our studies have found that drug brand responses can impact on drug selection and adherence (Wang et al., 2010; Cao et al., 2011). Besides, due to the brand recognition and expectation, when Chinese patients were switched from domestic drug (i.e., generics) to imported (joint venture) drugs, they usually reported a better drug efficacy (Ge and Zhu, 2007). However, as a vast, whether in unbalanced developed countries or in low-to-middle income countries, the medical resource is limited (Xu, 1990; Huo et al., 2016). We cannot offer all patients with imported (joint venture) drugs nor should we with good quality domestic drug. It is of importance to rational use the limited medical resource in China (Gao et al., 2011; Jin et al., 2015; Liu et al., 2016). Consequently, domestic drug prescribing is encouraged by all levels of government. In this work, we reported the efforts that encourage patients with brand response to use domestic drugs rather than imported (joint venture) drugs, aiming to provide some useful information for rational drug use.

## Patients and methods

### Patients

Patients with a diagnosis of mild-to-moderate depression in Fengxian Mental Health Center were recruited. The inclusion criteria included a requirement that patients have the ability of reading and understanding, have consciousness during the survey and are willing to participate in this investigation. The demographic information was collected including gender, age, education, course of treatment, and current drug application. The protocol was approved by Fengxian Hospital' Institutional Review Committee.

### Questionnaire

The questionnaire included brand responses and medication compliance. Brand response questionnaire consists of five questions (Wang et al., 2010): (1) You prefer to use domestic drugs because they are safe, effective, low side-effects, and economic? (2) You prefer to use imported (joint venture) drugs because they are safe, effective, low side-effects, and economic? (3) Do you think domestic drugs are equivalent to imported (joint drugs)? (4) Is it OK if you switch from domestic drugs to imported drugs? (5) Is it OK if you switch from imported drugs to domestic drugs? All questions have score standard: 2 for “agree,” 1 for “uncertain,” 0 for “disagree.” While the score of question 1, 3, and 5 was reversed. The total score points ranged from 4~0. Patients with 4 points were considered as brand response. Patients with 3~1 points were considered as low brand response. Patients with 0 point were considered as no brand response. The self-made questionnaire has good reliability and validity and had been used in China (Wang et al., 2010; Cao et al., 2011)

General medication compliance questionnaire was conducted according to compliance questionnaire (Chinese-version) (Zhao et al., 2002; Xie et al., 2013). The total score ranged from 0 to 11 points. Patients with a high score indicated better medication compliance.

### Drug switch

In order to observe the impact of drug switch from imported (joint venture) drugs to domestic drugs on safety and efficacy, patients who currently used imported (joint venture) drugs and displayed brand response (4 points) were recruited for further study. It is an open, quasi single-arm test without any parallel control but with self-control. The patients provided written informed consent (a few patients gave oral informed consent), and then were offered medical evidence on bioequivalence between imported drugs and domestic drugs, and explained the significance of pharmacoeconomics in this limited medical resource country. According to patients' condition and follow-up frequency, patients divided into three groups, were given domestic drugs to replace corresponding imported drugs for 8 weeks. The drug switch program was free of charge. During the switch period, physician follow-up was given once a week, 20 min for each time. In the follow-up course, patients were reviewed carefully, strengthened by rational drug use education and provided with psychological support. The safety and efficacy were evaluated before and after the 8-week switch. Adverse drug reactions were reported to investigators at any time.

### Safety/adverse reaction report

Adverse drug reaction was evaluated by Treatment Emergent Symptom Scale (TESS). Appropriate measures were ready to deal with any reaction if necessary.

### Efficacy

Hamilton Depression Scale (HAMD) was used to assess the efficacy (Lai et al., 2015). HAMD contains 24 items, most of the items scores from 0 to 4 point in 5 levels as follows: absent (0 point), mild (1 point), moderate (2 point), severe (3 points), and very severe (4 point). The total score of 0~7 is considered to be normal, 8~20 is suspected to have mild depressive symptom, 20~30 is considered to be depression, and >35 indicates severe depression.

### Statistical analysis

The SPSS for Windows 19.0 software (SPSS Inc., Chicago, IL, USA) was used for all data analysis. Quantitative variables were described as mean ± SD, while qualitative variables were expressed as number and percentage. Statistical significance was assumed at the *P* < 0.05 level.

## Results

### Patient demographic information and brand response

**Figure 1:**
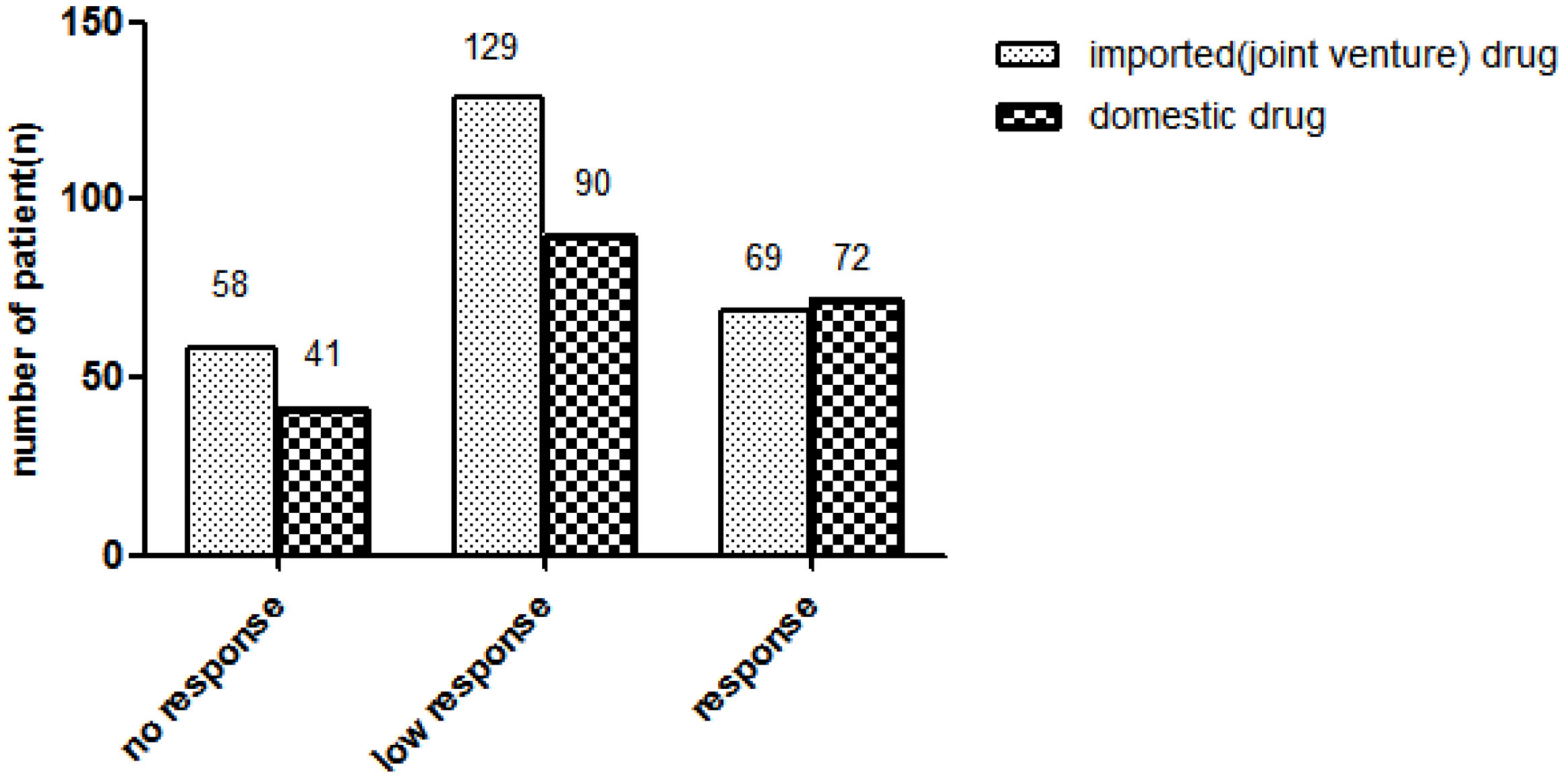
**Distribution of drug brand response in patients with depression**.

A total of 468 outpatients, aged between 12 and 80 years old, with a diagnosis of mild-to-moderate depression, were enrolled in the present study. Nine patients were excluded due to loss to follow-up. Finally 459 patients participated in and finished the program (Table 1). Sixty-nine of the 256 patients (27%) currently used imported (joint venture) drugs showed brand response. Seventy-two in 203 patients (35%) patients currently used domestic drugs showed brand response. No statistically significant difference existed between 2 groups for brand response (Figure 1).

**Table 1:** **Demographic data and brand preference in 459 patients**.

	**Total (*n* = 459)**	**Brand response (*n* = 141)**	**Imported drugs group (*n* = 256)**	**Domestic drugs group (*n* = 203)**
**GENDER**				
Female	236	75	142	93
Male	223	66	114	110
**AGE**				
<30 years	47	10	29	18
30–60 years	306	89	176	130
>60 years	106	42	51	55
**EDUCATION**				
Low	198	81	73	125
Middle	202	54	139	63
High	59	6	44	15
**ALCOHOL**				
Every day	2	0	1	1
Often	26	8	16	10
Sometimes	155	49	79	76
Never	276	84	160	116
**SMOKE**				
Yes	128	43	67	61
No	331	98	189	142

**Figure 2:**
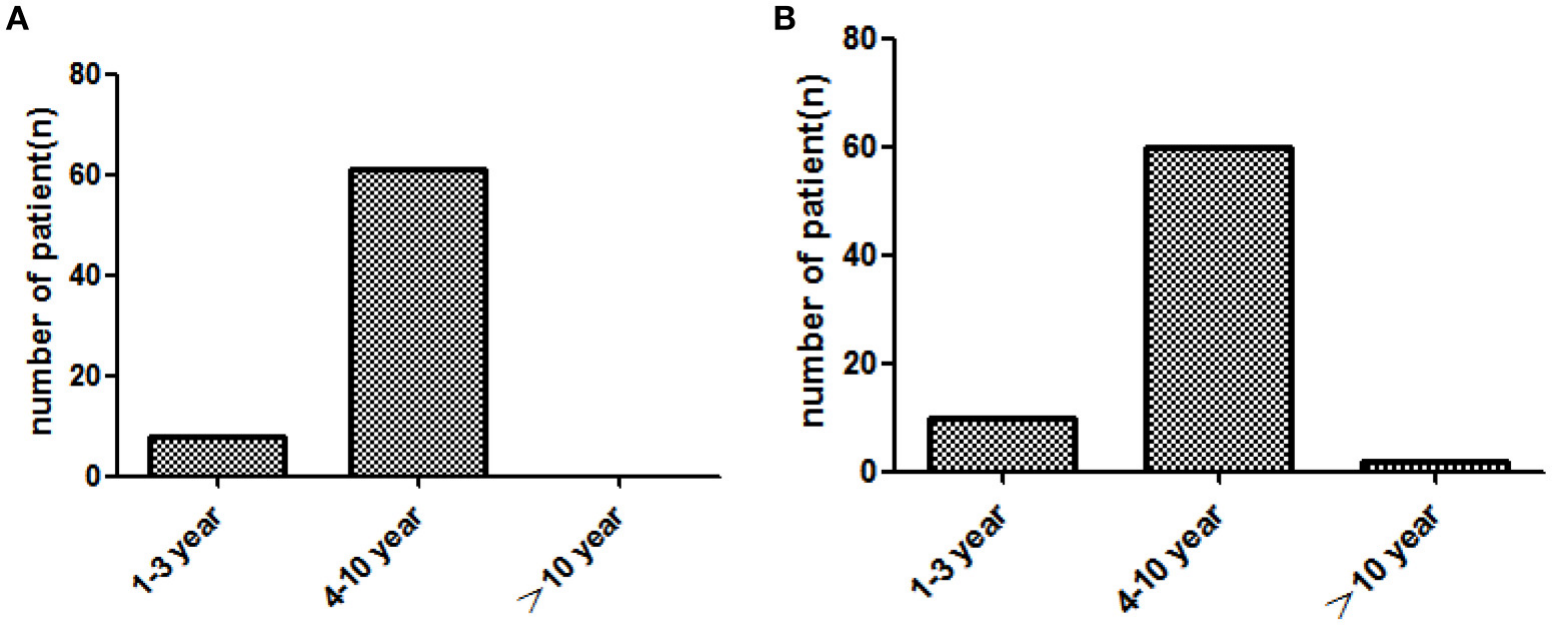
**Brand response with the different course of treatment (year) in two groups**. **(A)**: imported drug group, **(B)**: domestic drug group.

In two groups, patients displayed ascending brand response since treatment beginning, but gradually no longer response after a long time of medication (Figure 2).

### Brand preference and compliance

In general, the medication compliance of patients is acceptable. The compliance of patients in imported drugs group was similar to that in domestic drugs group (6.04 ± 2.08 vs. 4.74 ± 2.13, *P* > 0.05). Among the patients in imported drugs group, the compliance of patients with brand response was the best (6.30 ± 1.93). However, patients with brand response in domestic group did not have such optimal compliance (4.63 ± 1.94) (Table 2). Correlation analysis showed that the compliance was positively related with brand response in the imported drugs group. However, compliance was negatively related to age, course of treatment both in the imported drugs group and in the domestic drugs group. Yet compliance was related to education in domestic drugs group (Table 3).

**Table 2:** **Compliance in different brand response patients in the two groups**.

**Items**	**Imported drugs (*****n*** = **256)**			**Domestic drugs (*****n*** = **203)**		
	**No response**	**Low response**	**Response**	**No response**	**Low response**	**Response**
Compliance self-assessment	2.10 ± 0.61	2.16 ± 0.54	1.93 ± 0.60	2.15 ± 0.48	2.11 ± 0.61	1.97 ± 0.73
Forget to take drugs sometimes	0.43 ± 0.50	0.57 ± 0.50	0.74 ± 0.44	0.32 ± 0.47	0.28 ± 0.45	0.28 ± 0.45
Forgot to take drugs in the past 2 weeks	0.83 ± 0.38	0.88 ± 0.32	0.88 ± 0.32	0.88 ± 0.33	0.68 ± 0.47	0.61 ± 0.49
Self determine to reduce dosage or withdraw drugs w/o inform physicians	0.66 ± 0.48	0.71 ± 0.45	0.80 ± 0.41	0.56 ± 0.50	0.47 ± 0.50	0.68 ± 0.47
Take no drugs when travel	0.03 ± 0.18	0.04 ± 0.19	0.00 ± 0.00	0.00 ± 0.00	0.03 ± 0.18	0.06 ± 0.23
Forgot to take drug yesterday	0.03 ± 0.18	0.01 ± 0.09	0.03 ± 0.17	0.02 ± 0.16	0.06 ± 0.23	0.07 ± 0.26
Stop taking drugs by oneself as feel better	0.57 ± 0.50	0.73 ± 0.45	0.81 ± 0.39	0.56 ± 0.50	0.40 ± 0.49	0.54 ± 0.50
Feel difficult to adhere treatment for chronic diseases	0.40 ± 0.49	0.50 ± 0.50	0.54 ± 0.50	0.39 ± 0.49	0.29 ± 0.46	0.22 ± 0.42
Feel difficult to follow regimen	0.47 ± 0.50	0.55 ± 0.50	0.58 ± 0.58	0.37 ± 0.49	0.29 ± 0.46	0.19 ± 0.40
Total	5.52 ± 2.47	6.14 ± 1.94	6.30 ± 1.93	5.24 ± 2.28	4.60 ± 2.20	4.63 ± 1.94

**Table 3:** **Correlation between compliance with gender, age, education, brand preference and course of treatment**.

	**Compliance**	***P*-value**
**IMPORTED DRUGS GROUP**		
Gender	−0.06	0.31
Age	−0.22^*^	0.00
Education	0.12	0.05
Brand response	0.26^*^	0.00
Course of treatment	−0.24^*^	0.01
**DOMESTIC DRUGS GROUP**		
Gender	−0.01	0.94
Age	−0.26^*^	0.00
Education	0.34^*^	0.00
Brand response	0.04	0.60
Course of treatment	−0.24^*^	0.03

* *Correlation is significant at the 0.01 level (2-tailed)*.

### Drug switch efficacy

Sixty patients with brand response in imported drugs group were given rational drug use and pharmacoeconomics education. Their informed consent was obtained and they were provided corresponding domestic drugs (free of charge) for 8 weeks. Patients were divided into 3 groups according to the course of treatment. Before drug switch, patients' HAMD scores had no significant difference among the 3 groups. After 8 weeks of drug switch treatment, the HAMD score of 3 groups declined. However, the declines in patients in 2 groups with less than 10 years medication were statistically significant (*P* < 0.05). In general all patients with brand response continued to maintain good antidepressant activity when switched to domestic drugs switch (Table 4).

**Table 4:** **Efficacy (HAMD score) after drug switch in 60 patients with brand response**.

**Course of treatment**	**Before switch**	**After switch**	***P*-value**
1–3 years (*n* = 30)	17.67 ± 4.093	11.08 ± 5.030	0.000
4–10 years (*n* = 22)	15.69 ± 3.478	10.75 ± 5.447	0.013
>10 years (*n* = 8)	15.50 ± 1.000	11.00 ± 4.889	0.135
Total	16.75 ± 3.780	10.95 ± 5.058	0.000

### Safety and adverse drug reaction

During the 8-weeks domestic drugs switch treatment, suspicious or very slight adverse reactions were reported sporadically in 60 patients. Before switch, the main adverse reactions were thirst, constipation, sweating, insomnia, increased saliva, reduced activity, weight gain, nasal congestion, dizziness, and vertigo. After switch, the adverse reactions were almost the same. No moderate or severe adverse events were found during the switch.

## Discussion

Many patients display brand response both in high-earning patients and in low-income patients in China due to their blind brand worship and low health literacy (Cai et al., 2013; Xiao and Xu, 2014). Patients and their family members usually worry about safety, efficacy and quality of the generics. As to non-patient factors, the choice of imported drugs or domestic drugs has always been a challenge both in medication and in economics. Many physicians and hospitals relied on the profits from drug procurement for an appreciable proportion of their income—so not in their interests to prescribe/dispense low cost domestic drugs and persuade patients of the benefits of the imported (joint venture) drugs—leading to a strong placebo like effect(Reynolds and McKee, 2011; Li et al., 2012; Zeng et al., 2014). The competition between brand-name drugs and generics is becoming fiercer than ever before in the world, especially with the increase of newly approved generics in developing countries. Although domestic drugs (i.e., generics) have been verified bioequivalence to the originator (imported-joint venture drugs) in many published studies (Paton, 2006; Sakshaug et al., 2007; Kesselheim et al., 2008, 2010; Corrao et al., 2014a,b; Gagne et al., 2014; Lessing et al., 2014; Martin et al., 2014), quite a lot of patients in China still prefer brand-name drugs. Compared with the situation in China, foreign countries have different attitudes to generic drugs. In the Scotland, there are very high voluntary INN prescribing antidepressants exist (Godman et al., 2013a). In Sweden, the vast majority of physicians agree that they should prescribe generic drugs when available, meanwhile, they accept compulsory generic substitution including antidepressants as well (Andersson et al., 2006; Godman et al., 2013b).

Long term adherence is a real issue across products (Cramer et al., 2008). It is interesting to note that the medication compliance is almost the same regardless imported drugs use and domestic drugs use in this study, which is similar to many evidences. In fact, generics actually aid long term compliance as more economical in developed countries (Shrank et al., 2006; Simoens and Sinnaeve, 2014; Barbui and Conti, 2015). In United States the vast majority (83%) agreed that physicians should prescribe generic drugs when available, however, non-Caucasian has higher brand-name drug response than Caucasian and holds much skeptical attitude to the efficacy and safety of generics (Kesselheim et al., 2016). A survey conducted in Poland showed that about 3/4 of patients preferred to use generic drugs. Poland has one of the highest utilization rates for generics in Europe driven by domestic manufacturing and patients paying the price difference between the generic and a brand-name drug in a class (Drozdowska and Hermanowski, 2015). People's attitude to generics is often influenced by their physicians, pharmacists, family, friends, mass media and their health literacy. Pharmacists, as professionals, were more willing to use generics rather than brand-name drugs for self-management of common health problems due to their professional knowledge and health literacy (Patel et al., 2016). Unfortunately, there are a number of publications that have shown concerns with generics, which have impacted on their use (Van Ameringen et al., 2007; Margolese et al., 2010; Pae et al., 2010; Desmarais et al., 2011; Cessak et al., 2016).

The data in this work confirmed that about 30% of patients, no matter what class of drugs they use currently, have brand response. We also found that patient's brand response gradually declined as the treatment course extended. It may be due to the psychological fatigue induced by the long term medication course. Patients may not be as sensitive as at the beginning of treatment.

Medication compliance of patients in imported drugs group was positive correlated with brand response, however, no similar relationship occurred in domestic drugs group. The hidden reason for the difference is not clear. In general, medication compliance in the 2 groups was negatively correlated to age and course of treatment, suggesting we should pay more attention to elderly patients with long time medication.

According to our study, we found that as long as we provided rational drug use information, therapeutic equivalence evidences, pharmacoeconomics education and psychological support, patients even with brand response might be successfully switched to domestic drugs that might effectively save medical resource. In addition, providing the medicines free of charge might be one of the factors for the smooth switch, with published studies showing co-payments do influence the use of antidepressants (Barbui and Conti, 2015). Our results at least suggested that domestic drugs might replace the imported drugs with their acceptable safety and efficacy. Patients have right to choose domestics drugs or imported drugs depending on their economic situation, however, if our efforts help enhance the rational use of medical resource, the world will be better.

## Conclusion

Brand responses may benefit medication compliance in patients. With rational drug use education and psychological support, patients may be switched to generics to save limited medical resource in developing countries.

## Author contributions

Conceived and designed the study: FX. Performed the study: ML, JC, PZ, and CF. Analyzed the data: ML, JC. Contributed to questionnaire interview and data collection: PZ, CF. Wrote the paper: ML. Revised the manuscript: FX.

### Conflict of interest statement

The authors declare that the research was conducted in the absence of any commercial or financial relationships that could be construed as a potential conflict of interest.
